# Increased Stability of SARS-CoV-2 Omicron Variant over Ancestral Strain

**DOI:** 10.3201/eid2807.220428

**Published:** 2022-07

**Authors:** Alex Wing Hong Chin, Alison Man Yuk Lai, Malik Peiris, Leo Lit Man Poon

**Affiliations:** The University of Hong Kong, Hong Kong, China (A.W.H. Chin, A.M.Y. Lai, M. Peiris, L.L.M. Poon);; Centre for Immunology & Infection, Hong Kong (A.W.H. Chin, M. Peiris, L.L.M. Poon)

**Keywords:** COVID-19, coronavirus disease, SARS-CoV-2, Omicron, variant of concern, stability, severe acute respiratory syndrome coronavirus 2, viruses, respiratory infections, zoonoses

## Abstract

As of April 2022, the Omicron BA.1 variant of concern of SARS-CoV-2 was spreading quickly around the world and outcompeting other circulating strains. We examined its stability on various surfaces and found that this Omicron variant is more stable than its ancestral strain on smooth and porous surfaces.

The Omicron SARS-CoV-2 variant of concern (VOC) is highly transmissible in humans. As of April 2022, it has outcompeted other known variants and dominated in different regions (*1*). Its spike protein has >30 mutations compared with the ancestral strain (*2*). A 2022 structural study indicates the Omicron spike protein is more stable than that of the ancestral strain (*3*); this finding prompted us to hypothesize that Omicron VOC is also more stable on different surfaces. We previously showed that the ancestral SARS-CoV-2 strain can still be infectious at room temperature for several days on smooth surfaces and several hours on porous surfaces (*4*). 

We used previously described ancestral SARS-CoV-2 (PANGO lineage A) and Omicron VOC (PANGO lineage BA.1) in this study (*5*,*6*). We tested their stability on different surfaces using our previously described protocol (*4*,*7*). In brief, we applied a 5-μL droplet of each virus (10^7^ 50% tissue culture infectious dose [TCID_50_]/mL) on different surfaces in triplicate. We incubated the treated surfaces at room temperature (21°C–22°C) for different time points as indicated and then immersed them in viral transport medium for 30 min to recover the residual infectious virus. We titrated the recovered virus by TCID_50_ assays using Vero E6 cells, as described (*4*,*7*).

Compared with the ancestral SARS-CoV-2, the Omicron BA.1 variant was more stable on all surfaces we studied (Table). On day 4 postinoculation, we recovered no infectious ancestral SARS-CoV-2 from stainless steel, polypropylene sheet, or 2 of 3 glass samples. We did not recover infectious virus from glass on day 7. In contrast, infectious Omicron variant was still recoverable from all treated surfaces on day 7 postincubation.

**Table:** Stability of ancestral SARS-CoV-2 and of Omicron variant on different surfaces*

Material	Incubation time†	Ancestral SARS-CoV-2			Omicron variant	
		Mean log_10_(TCID_50_/mL) ±SD‡	% Reduction in viral titer		Mean log_10_(TCID_50_/mL) +SD‡	% Reduction in viral titer
Stainless steel	0	5.02 +0.39	NA		5.35 +0.18	NA
	3 h	4.21 +0.36	85.15		4.82 +0.23	69.78
	6 h	3.73 +0.10	95.80		4.62 +0.31	79.86
	1 d	2.99 +0.17	99.21		4.65 +0.17	80.28
	2 d	2.08 +0.11	99.91		4.51 +0.15	85.82
	4 d	§	>99.93		3.72 +0.12	97.72
	7 d	§	>99.93		3.58 +0.30	98.19
Polypropylene	0	4.85 +0.23	NA		5.43 +0.16	NA
	3 h	4.12 +0.19	81.72		4.65 +0.34	81.27
	6 h	3.53 +0.15	95.43		4.33 +0.14	92.34
	1 d	3.13 +0.34	97.86		4.45 +0.23	89.25
	2 d	2.01 +0.10¶	>99.86		4.34 +0.25	91.53
	4 d	§	>99.88		3.97 +0.19	96.48
	7 d	§	>99.88		2.95 +0.27	99.65
Glass	0	5.10+0.24	NA		5.65 +0.28	NA
	3 h	4.26 +0.05	86.79		4.90 +0.15	83.62
	6 h	3.69 +0.11	96.42		4.52 +0.13	93.20
	1 d	2.83 +0.13	99.49		4.20 +0.01	96.84
	2 d	2.14 +0.13	99.90		4.43 +0.29	93.87
	4 d	1.96 +0.00¶	>99.93		4.06 +0.16	97.64
	7 d	§	>99.93		3.76 +0.10	98.83
Tissue paper	0	4.70 +0.22	NA		5.21 +0.14	NA
	5 min	3.85 +0.28	84.98		4.64 +0.70	53.94
	15 min	2.12 +0.14	99.75		3.72 +1.22	72.99
	30 min	§	>99.84		2.92 +0.40	99.34
	60 min	§	>99.84		§	>99.95
Printing paper	0	5.21 +0.00	NA		5.34 +0.13	NA
	5 min	2.69 +0.16	99.68		3.26 +0.42	98.91
	15 min	§	>99.94		2.20 +0.33¶	>99.91
	30 min	§	>99.94		2.16 +0.36¶	>99.92
	60 min	§	>99.94		§	>99.96

*Tests were performed in triplicate. NA, not applicable; TCID_50_, 50% tissue culture infectious dose. †The samples were incubated at room temperature (21°C–22°C). ‡Vero E6 cells were used for titration of viable viruses. §All the triplicates were below detection limit of the TCID_50_ assay. ¶One or two out of three replicates were below detection limit of the TCID_50_ assay.

The stability of the Omicron variant was also higher than ancestral SARS-CoV-2 on porous surfaces, such as tissue paper and printing paper. On tissue paper, viable ancestral SARS-CoV-2 was no longer recoverable after a 30-minute incubation. However, we detected viable Omicron variant after a 30-minute incubation. On printing paper, we detected no infectious virus after a 15-minute incubation. In contrast, viable Omicron variant was recovered from 2 of 3 replicates after a 30-minute incubation.

To confirm our observations, we used transmembrane serine protease 2 (TMPRSS2)–expressing Vero E6 cells to titrate infectious virus particles recovered from treated stainless steel and printing paper (Appendix Table). On stainless steel, infectious ancestral virus was undetectable on day 10 postincubation, whereas viable Omicron variant was still recoverable. Similarly, no infectious ancestral virus was detected on printing paper after a 30-minute incubation, whereas we detected viable Omicron variant in 1 out of 3 replicates. Although the virus could be trapped in the porous materials and inefficiently recovered, our findings confirm that Omicron variant is more stable than its ancestral strain on surfaces.

We noted that the cell line used for virus titration can affect our findings. It has been reported that Omicron variant is less dependent upon TMPRSS2 for cell entry (*8*); therefore, we were not surprised that different cell lines led to different viral inactivation profiles. Nonetheless, results from both cell lines suggest that the Omicron variant is more stable than the ancestral strain. This observation is consistent with other recent findings (R. Hirose et al., unpub. data, https://www.biorxiv.org/content/10.1101/2022.01.18.476607v1). More evidence is needed to account for the increased transmissibility of Omicron variant. The virus’s stability on surfaces may be one factor and should be taken into consideration when recommending control measures against infection. A recent study revealed that an infectious dose as low as 10 TCID_50_ units could infect >50% of human study participants (*9*). Our findings indicate that Omicron variant has an increased likelihood for transmission by the fomite route; they may also indicate that the enhanced stability deduced from structural studies (*3*) and now demonstrated on different surfaces may be relevant for droplet or aerosol transmission of SARS-CoV-2. Of interest, stability of avian influenza A(H5N1) viruses has been shown to have an association with transmissibility of avian influenza virus between mammals by the airborne route, although the mechanisms underlying this association are not fully understood (*10*). Further studies on the stability of Omicron variant and its emerging subvariants in droplets and aerosols are warranted.

One limitation of our study is that the experiments were conducted in a well-controlled laboratory environment. Variations in environmental conditions would affect the rate of viral inactivation. Therefore, the time required for virus inactivation that we demonstrated may not reflect all real-life scenarios. In addition, the components of the viral droplet medium applied in this study were different from those of the respiratory droplets, which could also affect the stability of the virus. Nonetheless, our findings demonstrate that the Omicron variant is more stable than the ancestral SARS-CoV-2 on different surfaces, a finding that may be relevant for determining recommendations for public health measures to limit virus transmission.

Appendix Additional information about the difference in stability of Omicron variant and ancestral SARS-CoV-2.
