# Supplementary material for: Increased Stability of SARS-CoV-2 Omicron Variant over Ancestral Strain
Source: Emerg Infect Dis. 2022 Jul;28(7):1515–7. doi: 10.3201/eid2807.220428 (PMC9239870; doi:10.3201/eid2807.220428)
Supplement: Appendix — Additional information about the difference in stability of Omicron variant and ancestral SARS-CoV-2. [file 22-0428-Techapp-s1.pdf]

Article DOI: https://doi.org/10.3201/eid2807.220428

Increased Stability of SARS-CoV-2 Omicron Variant over Ancestral Strain

Appendix

Appendix Table. Stability of the ancestral SARS-CoV-2 and Omicron variant on stainless steel and printing paper, titrated with TMPRSS2-expressing Vero-E6 cells*

		Ancestral SARS-CoV-2		Omicron variant	
Materials	Time of incubation†	Mean $\log_{10}$($TCID_{50}$/mL)±SD‡	% Reduction in viral titer	Mean $\log_{10}$($TCID_{50}$/mL)±SD‡	% Reduction in viral titer
Stainless steel	0	5.15±0.17	NA	5.45±0.08	NA
	6 h	4.48±0.21	77.86	4.82±0.13	76.64
	1 d	4.07±0.23	91.35	4.58±0.21	85.95
	2 d	3.75±0.11	96.14	4.48±0.32	87.78
	4 d	3.20±0.14	98.89	3.89±0.11	97.24
	7 d	2.67±0.12	99.68	3.37±0.29	99.04
	10 d	§	>99.94	2.55±0.08	99.88
Printing paper	0	4.32±0.04	NA	4.62±0.48	NA
	5 min	2.92±0.27	95.53	3.64±0.31	90.95
	15 min	2.09±0.12¶	>99.40	2.27±0.19	99.65
	30 min	§	>99.57	2.02±0.10¶	>99.81
	60 min	§	>99.57	§	>99.84

*Tests were performed in triplicate.NA, not applicable; TMPRSS2, transmembrane serine protease 2.

†The samples were incubated at room temperature (21-22°C).

‡TMPRSS2-expressing Vero-E6 cells were used for titration of viable viruses.

§All the triplicates were below detection limit of the $TCID_{50}$ assay.

¶One or 2 out of 3 replicates were below detection limit of the $TCID_{50}$ assay.
